# “I Can’t Breathe”: Examining the Legacy of American Racism on Determinants of Health and the Ongoing Pursuit of Environmental Justice

**DOI:** 10.1007/s40572-022-00343-x

**Published:** 2022-03-04

**Authors:** Jennifer D. Roberts, Katherine L. Dickinson, Marccus D. Hendricks, Viniece Jennings

**Affiliations:** 1 Department of Kinesiology, School of Public Health, University of Maryland, College Park, MD 20742 USA; 2 Department of Environmental and Occupational Health, Colorado School of Public Health, University of Colorado Anschutz, Aurora, CO 80045 USA; 3 Department of Urban Studies and Planning, School of Architecture, Planning and Preservation, University of Maryland, College Park, MD 20742 USA; 4 Department of Public Health, Agnes Scott College, Decatur, GA 30030 USA

**Keywords:** Environmental racism, Health inequities, Syndemics, Environmental justice

## Abstract

**Purpose of Review:**

“I can’t breathe” were the last words spoken by Eric Garner (July 17, 2014), Javier Ambler (March 28, 2019), Elijah McClain (August 30, 2019), Manuel Ellis (March 3, 2020), and George Floyd (May 25, 2020). These were all African American men who died at the hands of police in the United States. Recently, police brutality has gained critical and overdue attention as one clear manifestation of systemic racism. However, historical and current policies related to a wide range of environmental hazards have exposed Black, Indigenous, and People of Color (BIPOC) to disproportionately high levels of physical, mental, social, emotional, and cultural toxicities, thus creating unbreathable and unlivable communities.

**Recent Findings:**

This paper traces the roots of systemic anti-Black racism in America from its origins in the 1400s, through systems of scientific racism that pathologized Blackness in order to justify slavery, and through evolving policies and structures that have shifted over time but consistently exposed many African American communities to unsafe and unhealthy environments.

**Summary:**

We conclude with calls for bold solutions to move through and past this oppressive history and toward true environmental justice the enables all communities to thrive together.

## Introduction

*This is racism. This is environmental racism* [1].

Benjamin F. Chavis Jr.

In 1982, Dr. Benjamin F. Chavis Jr., an African American civil rights leader, spearheaded a protest against the dumping of soil laced with polychlorinated biphenyls (PCB) in a poor area of Warren County, NC. Unlike many of the other protestors who were arrested for lying down in front of the dump trucks delivering the loads of hazardous waste, Chavis was hurled in jail by a North Carolina state trooper for driving “too slowly” when he traveled by car to bail out the protestors [2]. He then clutched the jail bars and retorted “This is racism. This is environmental racism.” [1]. While the disposal of PCB, a group of chemicals classified as human carcinogens, in an African American farming community was ostensibly what Chavis was referring to as “environmental racism,” his words, spoken while jailed for the crime of “driving while Black,” encompassed so many other forms, patterns, levels, and sides of racism that have persisted historically and contemporarily [3].

Beginning with the pilferage of African bodies, this article takes a journey from the earliest manifestations of scientific racism for the purpose of domination and colonization to the modern day repackaging of racism within the USA. Environmental racism, a phrase coined by Chavis and defined by Dr. Robert D. Bullard as “any policy, practice or directive that differentially affects or disadvantages (where intended or unintended) individuals, groups or communities based on race or color,” has disproportionately impacted the health and well-being of low-income communities and Black, Indigenous, and People of Color (BIPOC) over the course of generations [4]. Environmental racism has been well documented throughout the USA and is baked into the recipe of this country [5]. Moreover, the many forms of environmental racism that have been perpetuated over time have influenced the health inequities and disparities evidenced today. This review will present a historical examination of scientific racism, a focus on issues related to modern racism, and examine the realities of environmental racism as related to social determinants of health through an African and/or Black American lens. All three forms of racism will be exemplified through a discussion of the 1918 influenza pandemic and surrounding events as well as the coronavirus (COVID-19) syndemic of today. Finally, this review will end with calls for bold solutions to achieve environmental justice or rather “the fair treatment and meaningful involvement of all people regardless of race, color, national origin, or income with respect to the development, implementation and enforcement of environmental laws, regulations and policies,” and a declaration that “racism costs everyone” [6, 7].

## Scientific Racism

### “Stamped From the Beginning”

*Race creates new forms of power: the power to categorize and judge, elevate and downgrade, include and exclude* [8].

Ibram X. Kendi

Reaching as far back as the 1400 s, justifications for atrocities against African bodies have prevailed. These justifications in the form of scientific racism, or rather the pseudoscientific conception that empirical evidence confirms White biological superiority, have morphed throughout the centuries to undergird and maintain racial inequality. Scientific racism, by way of comparative anatomy, physical anthropology, or eugenics, has been one of the most effective tactics used to legitimize and propagate anti-Black racism and White supremacy [9, 10]. For example, during the seventeenth century, William Petty, an English scientist and philosopher, rose to prominence for his progressive and groundbreaking economic theories. However, as a founding father of racism, he was also credited for some of the earliest scientific racism theories [11, 12]. Petty contended that the races were not just distinguished by physical characteristics, but also “in their natural manners and in the internal qualities of their minds” [10]. In his manuscript, *Of the Scale of Creatures*, Petty maintained a hierarchical pyramid of humanity in which the White race was at the top and lesser creatures, such as worms and “Guinea Negroes” were at the bottom [10]. Later in the eighteenth century, David Hume, a Scottish philosopher and social theorist who influenced the development of skepticism as well as the experimental science of human nature, wrote an essay titled *Of National Characters*. This essay divided and ranked the human species intellectually and morally with statements like “in very barbarous and ignorant nations, such as the Africans and Indians” and “greater wit and gaiety in a Frenchman than in a Spaniard” [13]. Specifically, Hume positioned the African or Negro at the lowest rank and explicitly stated: “I am apt to suspect the Negroes to be naturally inferior to the Whites.” [13]. As just one more example, George Cuvier, a French naturalist and zoologist, conducted studies substantiating theories of scientific racism. He is most widely known for his dissection of Sara “Saartjie” Baartman, a South African woman brought to Europe in the nineteenth century under the ownership of William Dunlop, an English surgeon who used her as a domestic servant and for entertainment purposes [14]. Due to her physique, explicitly her buttocks and unique coloring, Baartman was exploited and exhibited as a freak show attraction. In 1815, Baartman died and Cuvier performed her “autopsy.” In his report, he noted that she had ape-like traits, compared her to an orangutan and stated “never [had he] seen a human head more resembling a monkey’s than hers” [10].

#### Medicalizing Blackness

As this scientific racism dogma prevailed in the newly forming USA, a preoccupation with Blackness and the construction of race was apparent in studies conducted from the seventeenth through early nineteenth centuries on tangible traits unique to Black bodies. These studies became the hallmarks of American medicine as Blackness continued to be defined as a marker of difference and racial inferiority [15, 16]. Thus, American physicians would constitute their discussions of racial difference with physiological proof, and the pathologizing of Blackness (e.g., smaller skulls, weaker lungs) provided a tool to separate, oppress, and exert power over Black bodies [17, 18]. Josiah C. Nott, a southern slave owner, was an American physician known for his work in obstetrics and gynecological surgery as well as his research in the etiology of Yellow Fever [19]. Nott, who began practicing medicine in 1829, co-opted the authority of science and medicine to defend the subjugation of African Americans through slavery [19]. He argued that “the Negro achieves his greatest perfection, physical and moral, and also greatest longevity, in a state of slavery” and went further to classify Negroes as “the lowest point in the scale of human beings” [19, 20]. Therefore, any race mixing with the White population was “insulting and revolting” [19].

#### Runaway Slave Syndrome

The creation of racial distinction and the medicalization of Blackness were interlinked and vital to the development of medical ideologies in the USA, and were also critical for the rationalization and sustainability of slavery. As the Civil War loomed in the mid-1800s, the two-centuries-old institution of slavery, which formed the fabric of America’s economic development and prosperity, was under threat. By its very nature, medical illnesses were conceived to uphold the pretext of slavery. Drapetomania, a “medical illness” exhibited by bondpeople when they absconded or even expressed and acted upon a desire for freedom, was invented by Samuel A. Cartwright [21, 22]. Cartwright, a practicing physician in Alabama, Mississippi, and Louisiana, believed that there were physical, mental, and physiological differences between the Negro and White races, which necessitated Negro bondpeople being controlled and coerced. By 1850, Cartwright designed a specialty in “Negro Diseases” and slave labor management by publishing the *Report on the Diseases and Peculiarities of the Negro Race* the following year. Cartwright also concocted dysaesthesia aethiopica, a disease causing “rascality” in bonded and free African Americans [22]. He shaped beliefs pertaining to Black bodies through his sentiments. It was Cartwright’s position that any radical behavior among Negroes should be attributed to mental illnesses and that his therapeutic cures would preserve the institution of slavery and serve the economic vitality of the American South and entire country.

### Emancipated, but not Free

After the American Civil War, enslaved people were declared free, granted citizenship, and extended the right to vote through the 13th, 14th, and 15th amendments, respectively. However, the period after the war, Reconstruction, was decisively sabotaged by the repackaging of racial oppression through sharecropping, Black Codes, and convict leasing. Consequently, African Americans were systematically impoverished, disenfranchised, terrorized, and marginalized from mainstream society.

#### Reconstruction and Beyond

Sharecropping was an agricultural system instituted in southern American states where former slave masters no longer had free labor to cultivate their land and millions of freed slaves were in search of work. As a result, it was common for former masters to offer their former bondpeople jobs by way of sharecropping. Importantly, though, these former bondpeople had no residence, farm equipment, or livestock and needed to purchase these resources on credit from the landowner in order to begin working. With a sharecropping contract, African American farmers were then required to give a portion of the crop yield, namely, shares, back to the landowner in lieu of paying cash for rent or the payback of farming supplies [23]. This sharecropping system kept African Americans in a perpetual and permanent state of poverty and unable to leave the farm until all debts were paid, a premise defined through Black Codes. In an effort to control the African American labor force and aver White supremacy, Black Codes were laws devised to restrict freedom, deny equality, and ensure cut-rate labor. Although Black Codes varied from state to state in the bygone Confederacy, all were intended to replace or replicate the social controls of slavery. Some states limited the type of property that African Americans could own and in most states there were vagrancy Black Codes, which declared “freedmen, free Negroes and mulattos” over the age of eighteen as vagrants if unemployed and without permanent residence [24, 25]. If a person was found in such a state, arrest, fine, and penal labor in the form of convict leasing were the repercussions.

**Fig. 1:**
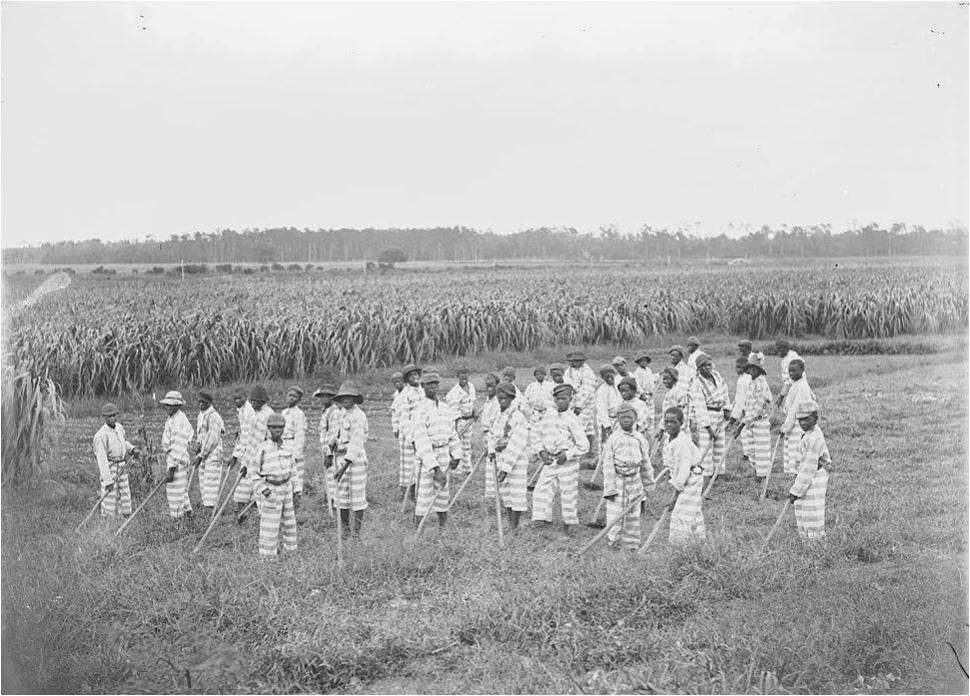
Juvenile convict leasing prison c. 1903. US Library of Congress Prints and Photographs Division, Washington, D.C. 20540, USA

Convict leasing was a system of forced manual labor. Under a contract with the county, the prisoner was sent to a private company prison and the county court was paid per prisoner and per month [26]. Convict leasing, often referred to as “slavery by another name,” became lucrative for judges, sheriffs, jurors, and the state counties each time a conviction and sentence was determined, often for arbitrary arrests and charges. As an example, in 1883, 10% of Alabama’s state revenue was derived from convict leasing, but by 1898 this amount increased to 73% of total revenue and over 85% of forced laborers were African Americans [26]. Prisoners, who were overwhelmingly African American men, were leased to labor on railways, plantations, and even in mines for sentences that were months or years [27]. Often these men and even boys were sent to the most dangerous locations because African Americans were considered expendable and the lessees sought to feed, clothe, and house the prisoners for a pittance of costs (Fig. 1) [28]. Workers were exposed to numerous environmental and occupational hazards, including violence and torture through beatings, communicable diseases (e.g., malaria, pneumonia, tuberculosis) from contaminated water and inhabitable shanties, as well as, treacherous working conditions and contaminants [29]. Commonly, prisoners, who worked on railroads were exposed to asbestos, which caused lung diseases and cancer, while the coal miners were exposed to crystalline silica dust, another respiratory toxicant, and the dangers of explosions [30, 31]. As such, in April 1911 approximately 128 men died in a Banner Mine explosion in Alabama while they were serving their penal term, the same state receiving over 70% of their total revenue from convict leasing [32].

#### The Great Migration

During Reconstruction, White supremacy was rampant and a reign of terror was maintained by groups like the Ku Klux Klan, which were often supported by state and local governments. Between 1882 and 1968, there were over 3,400 lynchings of African Americans and most occurred in the southern states [33]. As result of this Jim Crow South terrorism, along with the oppression of Black Codes, and poor economic conditions, including the sharecropping system and the ravaging effects of the boll weevil plague on cotton farming and production in the 1890s, many African Americans fled the South in what would be later called the Great Migration [34]. From 1915 to 1970, this massive internal exodus of approximately six million African Americans from the rural South to northern, midwestern, and western urban areas occurred in two phases, the First Great Migration (1915–1940) and the Second Great Migration (1940–1970) [35, 36]. This vast and leaderless watershed migration transformed urban America and significantly increased the share of African Americans in cities like New York, NY; Detroit, MI; Chicago, IL; Philadelphia, PA; and Los Angeles, CA. As an example, in 1910 the African American population in Gary, IN, was 2%, but by 1940 the share of African Americans reached 18%, the largest change during the First Great Migration [35]. Many migrants traveled by train with the help of African American porters and others fled the South by boat, bus, car, and in some cases horse-drawn carts [37]. Regardless of how they arrived all were lured by the anticipation of better employment and educational opportunities and simply the desperate need to experience the “warmth of other suns” as so eloquently stated by Richard Wright, a prolific American writer who wrote of his journey in the Great Migration [38]. Advertisements in African American newspapers, like the “Chicago Defender” or the “Urban League Bulletin,” touted higher wages (e.g., Henry Ford’s “Five-Dollar Day”) and northern living benefits, thus giving rise to the creation of a new African American city living culture [39–41].

#### Urban Living With a Color Line

Throughout the Great Migration, urban living for newly arriving migrants was overcrowded and quite inhabitable. A large portion of African Americans lived in cramped, dilapidated, and impoverished housing that brought about very unhealthy conditions [42]. Unsurprisingly, the mortality rates of African Americans were consistently and significantly higher than their White counterparts and the children were even more vulnerable to these poor living conditions [42–44]. To illustrate, in 1921 the mortality rate for African American and White infants was 108 and 72 per 1,000 live births, respectively [43]. Furthermore, an interesting difference in African American infant mortality fell along urban/rural and northern/southern lines. In the USA, the overall the urban infant mortality for African Americans exceeded the rural rate during the period of 1933–1935, yet a distinctive contrast was observed in northern (urban: 81.0, rural: 100.9) and southern (urban: 109.3, rural: 80.2) states. The unhealthy living conditions in American cities contributed to all the African American infant mortality rates, but there was more of a significant lack of childcare facilities in southern cities [43].

While employment opportunities in factories, mines, steel mills, or slaughterhouses afforded an opportunity for higher pay, the working conditions were often arduous, dangerous, and competitive. The industrial world landscape changed with the descent of southern African American workers by the thousands and consequently tensions arose between the newly arriving migrants and the existing northern White workers [45]. In the eyes of White workers, the African American migrants were the cause of low wages, below par working conditions, unemployed White men, and the leverage gained by labor unions with the surplus of workers [45]. This was especially true since many White employers surrendered to the need for African American workers despite the semantic of Black bodies being deviant or inferior among many White Americans of the time. Also, as the number of White workers leaving for World War I increased, it was inevitable that African Americans would be hired. A Carnegie Steel Company official stated “If it hadn’t been for the Negro at that time, we could hardly have carried on our operations.” [45, 46].

Even though legalized segregation was not as apparent in the North as it was in the South, racism and discrimination were nevertheless inescapable and pervasive, and were increasingly supported by explicitly racist policies. One of the most impactful of these policies was a practice known as “redlining.” In 1933, the Home Owners Loan Corporation was created as a federal government entity responsible for creating “Residential Security Maps” that rated the mortgage risk level of neighborhoods in American cities. “Low-risk” neighborhoods were given an “A” rating and depicted in green on a map, while those deemed highest risk were rated “D” and shown in red or redlined. Information used to determine a neighborhood’s rating included housing quality, recent sales, environmental nuisances (e.g., proximity to factories), and crucially, the neighborhood’s racial and ethnic composition. Appraisal methods followed the guidance of Frederick Babcock’s *Underwriting Manual*, which stated that, “The infiltration of inharmonious racial groups will produce the same effect as those which follow the introduction of nonconforming land uses which tend to lower the levels of land values and to lessen the desirability of residential areas.” [47]. Documents justifying ratings for specific neighborhoods reflected a clear-cut focus on race. As an example, the Denver, Colorado Whittier neighborhood was given a “D” rating with the following explanation: “This is a better Negro section of Denver and is one of the best colored districts in the USA. The northeastern part of it is often referred to as the ‘Negro Country Club’. […] Were it not for the heavy colored population much of it could be rated ‘C’.” [48]. This and other explicitly racist housing policies and zoning significantly limited African Americans homeownership and solidified racial housing segregation and wealth inequity across the country and across generations.

## Modern Racism

### “America Isn’t a Racist Country”

*Racism is not a big deal in America, Black people only make it a big deal because they are still upset about slavery, but that was in 1860* [49].

Salina, Kansas High School Student

As tempting as it may be to see the previously described racism as problems of the distant past, structural racism persists in the USA through cascading effects of past policies and the enduring impacts of modern “race-neutral” policies and ideologies. Paul Finkelman, a scholar of American legal history and race relations, states in his critique of Dinesh D’Souza’s *The End of Racism* that “In stepping away from scientific racism, the ‘new racism’ offers a cultural argument, lumping African Americans in a way that looks suspiciously like a repackaged version of the old racism.” [50]. Pointedly, African American culture, behavior, and civilization has merely been substituted for biology in the new or modern racism and continually denies the existence of discrimination or the need to consider race in any social policies [50]. Modern racists have denied the history of racial oppression in America and believe that racial equality has been achieved [51]. With such sentiments, downstream impacts of racism, such as inequities in housing, education, and other social determinants of health, have been consistently attributed to pathologies of African American culture and behavior rather than being seen as the effects of past and current policy. Thus, one of the issues faced today is how to position and impart health inequities in the context of racism.

#### Racism and Health

The Centers for Disease Control and Prevention (CDC) identified racism, or rather the norms that produce and normalize racial inequities, “a fundamental driver of racial and ethnic health disparities” [52, 53]. Racism, both interpersonal (e.g., racist biases that occur among or between individuals) and institutionalized or structural (e.g., cumulative and compounding racism embedded through laws, policies, and regulations within a society that systematically advantage White individuals and communities), has adversely impacted the mental and physical health of BIPOC [54, 55]. Furthermore, the deleterious forces of racism have been so pervasive and omnipresent that the effects have been embedded in all conditions of life, specifically throughout many social determinants of health.

#### Health Determinants

**Fig. 2:**
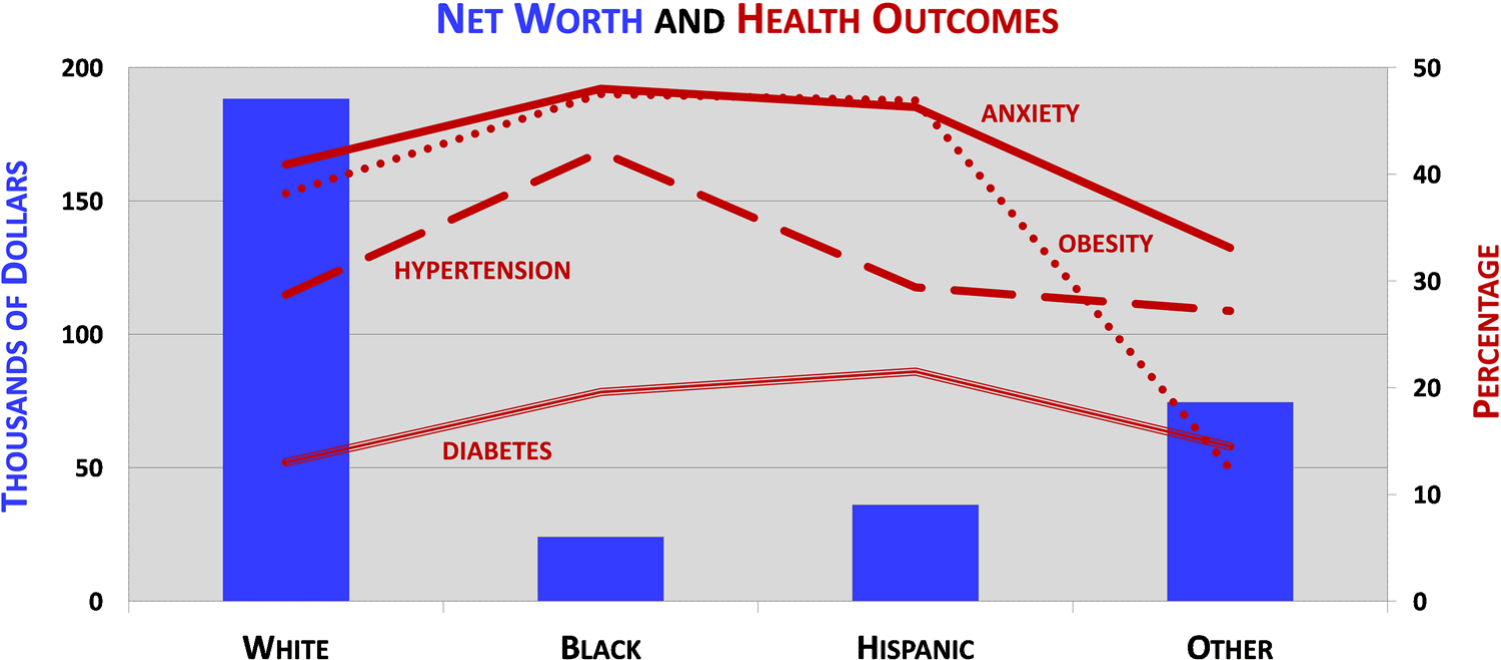
Relationship between wealth and health. Figure constructed using 2019 Federal Reserve Board (median net worth), 2019 Centers for Disease Control (diabetes, obesity, hypertension), and 2020 Kaiser Family Foundation (anxiety) data. Data available at Federal Reserve Board—https://www.federalreserve.gov/econres/notes/feds-notes/disparities-in-wealth-by-race-and-ethnicity-in-the-2019-survey-of-consumer-finances-accessible-20200928.htm#fig1; Centers for Disease Control—https://www.cdc.gov/nchs/hus/spotlight/HeartDiseaseSpotlight_2019_0404.pdf; and Kaiser Family Foundation—https://www.kff.org/coronavirus-covid-19/issue-brief/the-implications-of-covid-19-for-mental-health-and-substance-use/

“Your zip code is a better predictor of your health than your genetic code,” a pronouncement stated by Dr. Melody Goodman that recognizes the overwhelming variance of health and life expectancy among BIPOC individuals and communities in specific geographical neighborhoods due to social determinants of health [56, 57]. One way to understand this phrase is to examine all determinants of health [58]. All individuals are born and subsequently possess a set of individual health determinants that are related to genetic predispositions or generational influences for certain health outcomes and behaviors. Individual determinants can affect health behaviors (e.g., food intake), which can also affect health outcomes (e.g., obesity). These health outcomes can then turn back and affect health behaviors again or other health outcomes. Using COVID-19 as an example, certain health outcomes or preexisting illnesses, like diabetes or obesity, which often occur at disparate rates by racial and ethnic groups, have been shown to place individuals at a higher risk for severe COVID-19 illness and ultimately death (Fig. 2) [59]. Beyond individual determinants, an amalgamation of social determinants have also been shown to be even more impactful to heath behaviors and outcomes [60]. Everything from the built environment (e.g., housing and neighborhood) to the food environment (e.g., food apartheid) to the economic environment (e.g., jobs) can positively or negatively affect the health and well-being of individuals and communities (see Fig. 1 in Roberts et al. [58]). Again, turning back to COVID-19 as an example, research has found that many social determinants, such as housing quality, living conditions, occupation, income, transportation, and race, were all correlated with COVID-19 incidence and mortality [58, 61]. These social determinants and many others fall into environmental silos (e.g., built, food, economic, education, social, healthcare) that have been assembled, arranged, developed, and managed by laws, policies, and regulations born out of racist and discriminatory ideologies and practices. Therefore, it is inevitable that health and well-being is and has been patterned by race and place.

### America of Today Is America of Yesterday

In order to fully appreciate the intricacies surrounding contemporary issues of race and health, particularly among African Americans, the legacy of racists laws, policies, regulations, and even norms begs for a profound understanding. Everything from redlining and blockbusting to urban renewal have had a lasting effect on where and how African Americans live and the health inequities and disparities they experience. In the USA, access to quality goods and services have varied significantly along racial and income lines in the form of residential segregation sanctioned by local, state, and federal governments [57].

#### Residential Segregation

**Fig. 3:**
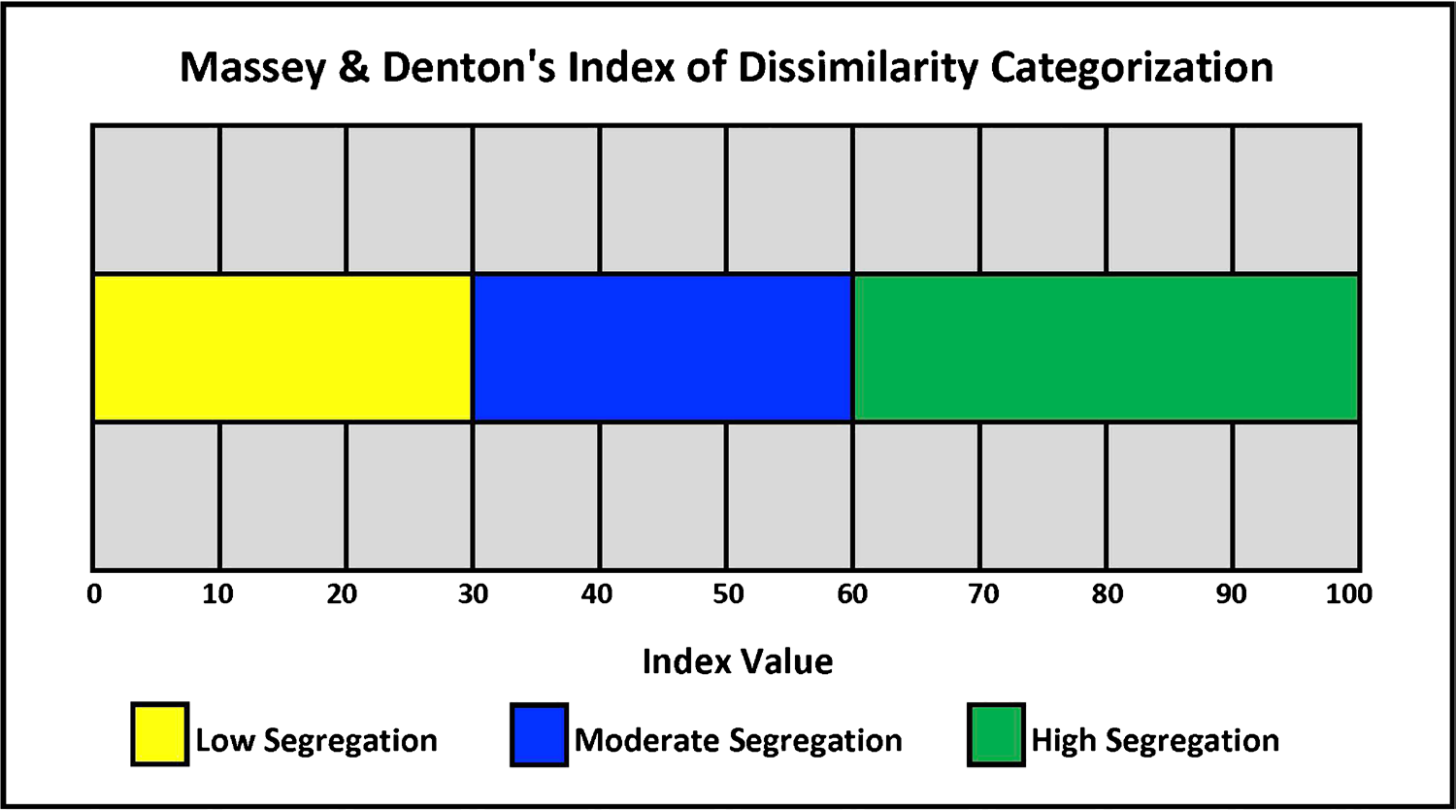
Index of dissimilarity categorization

Residential segregation in the nineteenth century was by census block, the lowest level among the standard hierarchy of census geographic entities, but by the next century this type of segregation elevated to the neighborhood level (e.g., block group or above) whereby affluent White and poor African American families were separated by larger geographic domains [62, 63]. In 1900, the majority of African Americans lived in a few southern states and the dissimilarity index, a metric for measuring residential segregation between African Americans and Whites, was 64 between states and 69 between counties [64]. With an index of 0 indicating a complete integration of two groups and an index of 100 indicating complete segregation, these values of 64 and 69 demonstrated a lower level of high segregation (Fig. 3) [63]. However, by 1940, city level segregation sharply increased with indices over 80 (e.g., 86 Cleveland, OH; 82 Buffalo, NY), a function of the Great Migration and redlining policies whereby African Americans were living in highly segregated urban areas [64]. To state this phenomenon in another way, over 80% of African Americans in these cities would have had to move from their neighborhood to another one for the city to have become totally desegregated. Ramifications of suburbanization, along with White Flight, and residential segregation led to disinvestment and poverty in neighborhoods predominately inhabited by African Americans as well as the segregation of opportunities, such as good housing, schools, jobs, parks, transit, and many other social determinants of health that have contributed to health inequities and disparities [65].

#### Health and Place Inequities

The downstream legacies of racism in housing practices and city planning, which shaped residential settlement patterns, have been well documented for decades [66–68]. Specifically, a connection between redlining and health has been identified in hyper-segregated neighborhoods throughout the USA. Research examining the economic and health impacts of redlining in 142 urban neighborhoods (e.g., Rochester, MN; Richmond, VA) found statistically significant associations between redlined neighborhoods and higher rates of asthma, diabetes, hypertension, obesity, and kidney disease [69]. Furthermore, a lower life expectancy was also observed among neighborhoods with greater historic redlining. At one point, property and homeownership was the primary mechanism that families used to build and inherit wealth in America. And in general, housing equity is roughly two-thirds of all wealth for the median household [70]. Yet, when these provisions of wealth building were denied to African Americans by way of racist housing policies and practices (e.g., restrictive covenants; blockbusting), redlining and discriminatory lending, the existing wealth gap born out of the institution of slavery between African American and White families swelled enormously for decades [70, 71]. Furthermore, an inverse relationship between good health and wealth was observed with several health outcomes across racial and ethnic lines. Presently, the median White family holds nearly eight times the wealth of the median African American family and approximately five times that of the median Hispanic family [72]. And, when comparing 2019 Federal Reserve Board (Survey of Consumer Finances) median net worth data with 2019 CDC and 2020 Kaiser Family Foundation health outcome data by race and ethnicity, the African or Black American and Hispanic families experience higher rates of chronic disease and an inverse relation between wealth and health is observed (Fig. 2) [72–74]. In addition to health outcome disparities, inequities in neighborhood features, like parks and green space, have also been recognized. As grade “D” redlined neighborhoods were deemed hazardous and subsequently disinvested and neglected, grade “A” green neighborhoods were invested heavily and beautified with the planting of trees and creation of parks and parkways [75, 76]. In consequence to these inequitable investments, today African American (68%), Hispanic (67%), and Asian (67%) communities are nearly three times more likely that White (23%) communities to live in “nature deprived” areas or those with fewer forests, streams, and other natural places [77]. A preexisting issue that became more apparent and resounded during the 2019 COVID-19 pandemic [78, 79].

### Syndemic Tales in America

For many, the COVID-19 pandemic was seen as an “unprecedented” global calamity that intersected with and drew renewed attention to persistent racial and ethnic inequities in the USA [80]. In many ways, however, this “syndemic” is eerily similar to the 1918 influenza pandemic, with both events illustrating the cumulative effects of systemic racism on overlapping environmental and social health determinants during profound and life changing times of crisis [81].

#### 1918 Influenza Pandemic

Shortly after the First Great Migration picked up momentum and in the midst of World War I, the 1918 influenza pandemic erupted. It is believed to have been caused by a H1N1 subtype of an influenza A virus with avian and swine genetic origins and to have originated from Fort Riley, KS; Etaples, France; or Shanxi, China [82]. There were three distinctive waves and arguably a fourth minor wave that was localized throughout areas of New York City from Spring 1918 to Winter/Spring 1919 [81]. In total, the pandemic took the lives of over 675,000 Americans [81]. Much of the death rate was attributed to the unique W-shape curve. Traditionally, the curve of human influenza deaths has been U-shaped, with peaks among the most vulnerable, infants and older adults. However, this 1918 influenza pandemic exhibited a W-shape curve with the addition of a third peak among young adults from 15 to 34 years [81]. In addition to the significant increase in age-related deaths, the pandemic exacerbated the existing health disparities and inequities that most African Americans and other marginalized populations were experiencing. In cities, such as Baltimore, MD and Detroit, MI, tuberculosis became one of the top three causes of death among urban African Americans primarily due to the housing conditions. Individuals with tuberculosis lung damage were more susceptible to influenza, which resulted in a higher incidence of influenza-related pneumonia and subsequently higher case fatality rates during the second wave for African Americans [81].

**Fig. 4:**
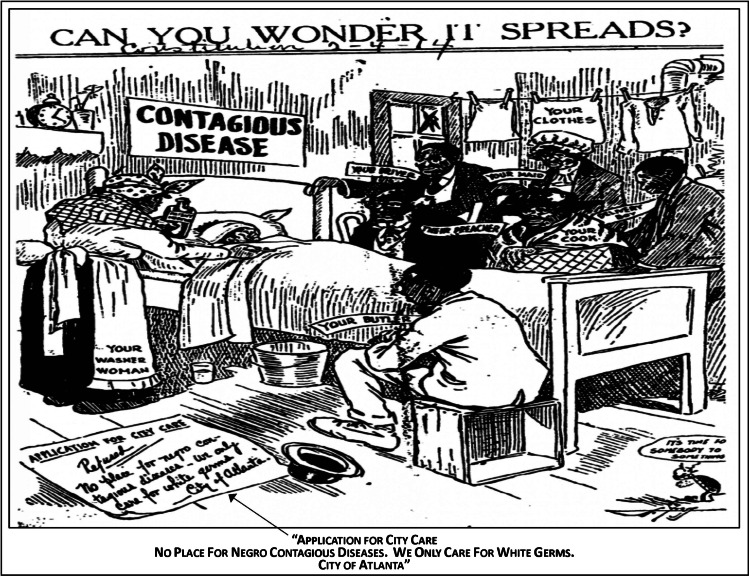
Atlanta Journal Constitution newspaper comic c. 1914

While segregated African American neighborhoods throughout urban centers served as ready-made quarantines, the entrenched housing and healthcare discrimination increased the influenza transmission, susceptibility, and fatality for African Americans. Access to healthcare professionals and facilities was restricted due to racism and legalized segregation. Even when care was received within segregated hospitals, African Americans received substandard service, often due to the overwhelmed capacity of hospitals and the heavy burden on medical staff. In some urban areas, like Philadelphia, PA, the limit of the Frederick Douglass Memorial Hospital, a hospital designated for African Americans, was exceeded and an “emergency annex” was erected in an African American parochial school. In Richmond, VA, African American patients received care in a hospital basement while the only African American facility in Baltimore, MD, was criticized for turning patients away. Therefore, many infected people died without seeing a doctor. Massive gravesites were dug to accommodate the rapidly growing number of corpses around the nation, particularly for African Americans who were often turned away from funeral homes and cemeteries. The 1918 pandemic amplified existing prejudices and scientific racism against African Americans, including myths of them being “diseased” with smaller skulls, weaker lungs, and impervious to pain [83]. These spurious notions of the biological and moral inferiority of African Americans were perpetuated by White supremacist ideologies and racist scientific theories. Furthermore, African Americans were often blamed for the spread of influenza during the pandemic. An example, printed in an Atlanta Journal Constitution newspaper comic, implied that “Negro contagious diseases” were spread to the White population by their cooks, delivery boys, maids, and other African Americans they employed (Fig. 4) [84]. Furthermore, these fallacies were disseminated by other contemporary newspapers with “Rush of Negroes to City Starts Health Inquiry”; “Negros Arrive by Thousands—Peril to Health”; or “Negro Influx Brings Disease” headlines [81, 85]. Even in the midst of a pandemic that took thousands of lives, the morality and humanity of mankind continued to deteriorate and decline.

#### The Red Summer of 1919

With the growing economic and social tensions surrounding the Great Migration, the sickness and death of the influenza pandemic, and the visceral anti-Black racial prejudices, a combustion of rage came forth in the summer of 1919. Named “Red Summer” for the amount of blood shed by James Weldon Johnson, the National Association for the Advancement of Colored People field secretary, lynchings, massacres, and riots occurred from April to November 1919 throughout the nation [86]. The July 27, 1919 drowning of Eugene Williams, an African American teenager who was stoned by a White mob after violating the unofficial Lake Michigan beach segregation line, caught the nation’s attention. Meanwhile, other White civilians and veterans in some of the nation’s most populous cities also formed mobs against African American residents. Some of the most violent episodes occurred in Chicago, IL; Washington, DC; and Elaine, AR and many incidents that occurred throughout the summer and beyond were exacerbated because government officials hesitated in taking action or turned a blind eye to the violence [87]. In total, hundreds of lives were claimed and over 1,000 African American families lost their homes and peace of mind to White mobs and rioters in this Red Summer of 1919 [86, 87].

While summer 1919 was a watershed moment of race riots, mob violence, lynchings, and White supremist terrorism, massive anti-Black brutality proceeded and succeeded the summer. Prior to the Eugene Williams murder, which signified the Red Summer of 1919, riots occurred in Jenkins County, GA (April 1919); Charleston, SC (May 2019); Longview, TX (July 1919); and Washington, DC (July 1919) [86]. Succeeding the summer memorialized by a Claude McKay poem, “If We Must Die,” the barbarity and savagery continued to escalate. In the late hours of September 30, 1919, sharecroppers gathered in a small church in Elaine, AR, a hometown of author Richard Wright (see “The Great Migration” section), to meet with a prominent White attorney from Little Rock, by the name of Ulysses Bratton, on the matter of unfair profits that the farmers were receiving from the White landowners. The meeting had been in session nearly 2 h when shots were fired into the church by a group of local White men. Shots were returned, resulting in one White man, Detective Will Atkins, being killed and rumors spread quickly that the sharecroppers were leading an insurrection. As a result, the mayors of Elaine and nearby Helena, AR, authorized groups of armed White men and local vigilantes to “hunt Negroes” and the governor of Arkansas called for over 500 soldiers from Camp Pike to “shoot to kill any Negro who refused to surrender immediately” [88]. With indiscriminate killing, at least 200 African American men, women, and children died. Years later and nearly 400 miles northwest in Tulsa, OK, one of the most surreptitiously hidden, albeit deadliest, massacres in American history, occurred. Following an all too familiar “Black man ‘hurt’ White woman” scenario that has been played repeatedly throughout history, a 19 year old African American man, Dick Roland, was arrested on May 30, 1921, after riding an elevator alone with a White woman named Sarah Page. With the story breaking in the Tulsa Tribune under the headline “Nab Negro for Attacking Girl in an Elevator,” tensions and anger swelled. African American Tulsans had every reason to believe that Dick Rowland would be lynched and were determined to protect him. Meanwhile, White lynch mobs grew and violence erupted. By the morning of June 1st, White rioters destroyed the section of the city known as Greenwood, a district recognized nationally for its affluent African American community. The commercial section of the district, including 191 businesses, was destroyed, along with 1,256 houses, a junior high school, several churches, and the one hospital [89]. While the estimated death figure runs well into the hundreds, to this day the number of deaths are unknown [86].

#### 2019 Coronavirus Pandemic

In late 2019, COVID-19, a novel coronavirus, emerged in China and spread into a global pandemic by the Spring of 2020. As of early 2022, COVID-19 had claimed the lives of over 850,000 Americans [90]. Immediately and throughout the pandemic, communities of color were particularly hard hit, with 178 deaths per 100,000 for African Americans, 172 for American Indians and Alaska Natives, and 154 for Hispanic/Latino Americans compared to 124 per 100,000 for White Americans [91]. Throughout the country, the percentage of African American COVID-19 vaccinations has been disparately lower to the percentage of African American deaths, a trend that is distinctly opposite of White Americans [92, 93]. And yet again, racist explanations for these disparities and for the spread of the pandemic have proliferated. Echoing past theories of scientific racism and biological differences between the races, some have posited that racial differences in mortality are due to genetic differences in lung function between African American and White populations [18, 94, 95]. Others blame behaviors, such as when Ohio State Senator and emergency room doctor Stephen Huffman mused: “Could it just be that African Americans – or the colored population – do not wash their hands as well as other groups?” [96]. Or when US Surgeon General Jerome Adams implored communities of color to “step up” and “avoid alcohol, tobacco, and drugs,” adding: “Do it for your abuela, do it for your granddaddy, do it for your big momma, do it for your pop-pop. We need you to understand, especially in communities of color.” [97]. Meanwhile, Asian populations have been blamed for the outbreak and spread of the pandemic, fueling racially motivated hate crimes against Asian Americans and Pacific Islander communities. In ways that mirror the 1918 pandemic, these racist fallacies have obscured the persistent role of structural racism, policy, and social determinants as true causes of racial disparities in health outcomes [83].

#### The Racial Reckoning Summer of 2020

In ways similar to and different from 1919, the summer of 2020 brought together a global pandemic and a reckoning over the persistent power of racism in America. On May 25, 2020, Minneapolis police officer Derek Chauvin murdered George Floyd by kneeling on his neck for more than 9 min while a crowd of people, including multiple children, watched and pleaded with Chauvin to heed Floyd’s repeated cries of “I can’t breathe.” This public and unabashed extrajudicial killing recalled public lynchings from a century earlier, and video footage of Floyd’s murder sparked a global uprising, with millions of people emerging from COVID-19 induced lockdowns to take to the streets and proclaim that Black Lives Matter. The movement was notable for its size and for its multiracial makeup. An estimated 15–26 million people participated in these protests, making this the largest movement in the country’s history [98]. After centuries of racial terror and systemic inequity, calls for change were louder and seemingly resonated more than ever, launching police reform initiatives across the country and landing “Racial Equity” as one of the four pillars of President Joe Biden’s campaign. At the same time, however, a smaller but vocal and powerful countermovement was underway. Protests organized purportedly against mask mandates and other government restrictions to control the COVID-19 pandemic also harnessed White supremacist forces, with some protesters waving Confederate and Nazi flags [99]. This movement’s virulence and violence, which became apparent when militias stormed the Michigan State Capitol in April 2020 to kidnap Governor Gretchen Whitmer, culminated in the US Capitol insurrection on January 6, 2021, an attempt to overthrow the 2020 Presidential election results [100]. The push and pull of racial progress and resurgence of White supremacy were on full display amidst the heightened tensions of the COVID-19 pandemic.

## Environmental Racism

### “An Old Wine in a New Bottle”

*Environmental racism, therefore, is a new manifestation of historic racial oppression. It is merely [an] old wine in a new bottle* [101].

Deborah M. Robinson

While Chavis and Bullard coined and defined environmental racism, Dr. Deborah M. Robinson relegated it as “an old wine in a new bottle” and to simply mean that environmental racism was a modernized materialization of historic racial oppression, subjugation, marginalization, and disenfranchisement [101]. The COVID-19 pandemic along with the Racial Reckoning Summer of 2020 was a reminder of how racism permeates every system and institution within the USA and how populations experiencing environmental injustice were vulnerable to many other public health issues. To illustrate, people with pre-existing conditions and who reside in communities with historically higher levels of air pollution were linked with more deaths from COVID-19 [102]. Likewise, an empirical study found that structural racism was a key driver in the racial disparities observed among COVID-19 cases throughout the USA [83, 103]. Over time and throughout this country, the many forms and interlocking factors of environmental racism have created a mosaic of varying hazards for many communities including, but not limited to, contaminated water (e.g., Flint Water Crisis [104, 105]), police brutality (e.g., Rodney King Beating [106]), toxic air (e.g., Cancer Alley [107–109]), racial profiling (e.g., Trayvon Martin [110, 111]), and food insecurity (e.g., Chicago Food Apartheid [112–115]) (Table 1). Despite these varying hazards, there has always been one constant with environmental racism; BIPOC have been on the receiving end of these harms and negative impacts in differing ways and extent, but collectively with little to no response from society at large.

**Table 1:** Examples of environmental racism in the USA

Incident	Year(s)	Hazard	Location	Description
Cancer Alley	1960s–present	Petrochemicals	Louisiana	Cancer Alley, an 85-mile stretch of land located in Louisiana along the lower Mississippi River between Baton Rouge and New Orleans, was originally named “Plantation Country” because it was home to a cascade of plantations where enslaved Africans were forced to labor. Today, Cancer Alley, a predominately African American area, is known for its pollution emitting, carcinogenic chemical plants, including 150 oil refineries, plastics plants, and chemical facilities. Research found that in Cancer Alley, 46 individuals per one million were at risk of developing cancer over a lifetime exposure to all carcinogenic air toxics in ambient air, compared with the national average of 30 individuals per one million.^1^
Rodney King Beating	1991	Police brutality	Los Angeles, CA	On March 3, 1991, White police officers were caught on video brutally beating an African American motorist named Rodney King during his arrest for driving while intoxicated. A bystander, George Holliday, filmed the incident from his nearby balcony and sent the footage to a local news station. Four officers were tried on charges of excessive force, but three were acquitted and a hung jury for the fourth officer. Immediately following the acquittals, the 1992 Los Angeles riots erupted, lasting for 6 days, and resulting in more than 50 deaths and 2,000 injuries.^2^
Trayvon Martin Shooting	2012	Racial profiling	Sanford, FL	Trayvon Martin was a 17 year old African American boy fatally shot on February 26, 2012, by George Zimmerman, a self-appointed neighborhood watch volunteer who called 911 to report a “suspicious person.” Trayvon was walking home from a trip to a convenience store. While George Zimmerman was charged with second-degree murder, he was subsequently acquitted by a six-woman jury on July 13, 2013. Trayvon Martin’s death and the trial of his murder gave rise to the Black Lives Matter movement.^3^
Flint Water Crisis	2014–2019	Lead	Flint, MI	A public health crisis occurred in Flint, MI, a majority African American city with 40% in poverty, when the local government switched its drinking water supply from the Detroit, Michigan system to the Flint River and failed to apply corrosion inhibitors to the water. This resulted in elevated lead exposure to approximately 100,000 residents.^4^
Chicago Food Apartheid	Present	Food insecurity	Chicago, IL	Food apartheid, the result of systematic racism and oppression, discriminatory policies in the form of zoning codes and lending practices, as well as overall systemic disinvestment in BIPOC, most often occurs in African American and Hispanic communities. Chicago, IL, is one of the worst and largest areas of food apartheid where there is a lack or absence of large grocery stores that sell fresh produce and healthy food options. Conversely, these area often have an abundance of fast food options, liquor stores, and corner shops that offer foods with high salt, fat, sugar, and other unhealthy ingredients.^5^

^1^James, W., Jia, C., & Kedia, S. (2012). Uneven magnitude of disparities in cancer risks from air toxics. *International Journal of Environmental Research and Public Health*, 9(12), 4365–4385. https://doi.org/10.3390/ijerph9124365

^2^NPR (2017). When LA erupted in anger: a look back at the Rodney King riots. Available at: https://www.npr.org/2017/04/26/524744989/when-la-erupted-in-anger-a-look-back-at-the-rodney-king-riots

^3^CNN (2021). Trayvon Martin shooting fast facts. Available at: https://www.cnn.com/2013/06/05/us/trayvon-martin-shooting-fast-facts/index.html

^4^NPR (2016). Lead-laced water in Flint: a step-by-step look at the makings of a crisis. Available at: https://www.npr.org/sections/thetwo-way/2016/04/20/465545378/lead-laced-water-in-flint-a-step-by-step-look-at-the-makings-of-a-crisis

^5^National Geographic (2021). Grassroots efforts take on ‘food apartheid’ in Chicago’s south side. Available at: https://www.nationalgeographic.com/history/article/grassroots-activists-take-on-food-apartheid-in-chicagos-south-side?loggedin=true

### “I Can’t Breathe”

“I can’t breathe” were the last words spoken by Eric Garner (July 17, 2014), Javier Ambler (March 28, 2019), Elijah McClain (August 30, 2019), Manuel Ellis (March 3, 2020), and George Floyd (May 25, 2020). While these African American men died as a result of police brutality, the hazards of breathing in toxins from other environmental domains of society are very apparent. Thus, environmental racism goes beyond the pollutants in air, water, food, or other environmental medium. Environmental racism spans to various dimensions of marginalization that diminish the social fabric of society. As noted in the Healthy People 2030 framework, racism and environmental conditions are key factors within social determinants of health [116]. This framework acknowledges the pronounced and interconnected role of determinants that impact public health. Strained social conditions, such as poverty, violence, or racial profiling, can also impress the interest and opportunity to engage in health promoting behaviors. For instance, a study among low-income communities in New York City found that park use was negatively linked with stop and frisk incidents [117]. Environmental racism can also relate to the availability of green spaces (e.g., parks, tree cover) in underserved areas. Reoccurring themes of limited access to quality green spaces in racially and ethnically diverse communities has also overlapped with health disparities related to heat stress, obesity, and cardiovascular disease [118]. Along with the availability of amenities, environmental racism can reinforce burdens to environmental health. For example, energy burden pertains to the household cost on energy utilities compared to its gross income [119, 120]. A study on energy burden, social capital, and environmental health found that counties with a higher proportion of African American residents showed statistically higher rates of premature mortality and lower life expectancy [120]. Likewise, counties in the USA with low to moderate income experienced greater energy burdens and poorer health [120]. This energy burden research detangled factors related to race, ethnicity, and income disparities in its analysis. Similar to the vision of environmental justice, the individual roles of race, ethnicity, and income can provide various implications on the population considered. Others have noted how continued cases of environmental racism led to higher lead exposure in private areas predominately occupied by African American residents [121]. Furthermore, Nigra discussed how racism has infiltrated the regulatory and reporting practices related to the Lead and Copper Rule. Along with water quality, social inequalities have led to physical infrastructure and natural disaster vulnerabilities. Underserved populations are more likely to experience greater damage, injuries, mortality, and slower recovery rates following disasters [122]. Collectively, environmental racism has hindered the opportunity for BIPOC to experience health equity and the chance to achieve optimal health.

### Achieving Environmental Justice

Environmental racism is and has been the vehicle to establish, advance, and perpetuate anti-Black racism and other forms of oppression brought upon marginalized communities. Since the inception of this country, racism has moderated outcomes within the natural, built, and social environments. Now in light of the COVID-19 pandemic, the virtual environment can also be added to this list. Many factors that drive environmental racism also fuel other inequalities. As noted in its definition, systemic racism exists in different systems beyond the field of environmental health. In fact, environmental racism sets the stage for just about every other form of inequality. Whether we consider the injustice of over-policing, crumbling infrastructure, over-polluting, elevated COVID-19 risks, climate impacts, food desserts, unsafe housing, or houselessness, environmental racism is the man-made construct driving it all. On October 24–27, 1991, in Washington, DC, delegates to the First National People of Color Environmental Leadership Summit drafted and adopted 17 principles of environmental justice. Since then, these principles have served as a set of guiding values for the revolutionary grassroots environmental justice movement that by most accounts began in Warren County, NC, as referenced previously. An introduction to the 17 principles prepared by the First National People of Color Environmental Leadership Summit reads as follows:

“WE, THE PEOPLE OF COLOR, gathered together at this multinational People of Color Environmental Leadership Summit, to begin to build a national and international movement of all peoples of color to fight the destruction and taking of our lands and communities, do hereby reestablish our spiritual interdependence to the sacredness of our Mother Earth; to respect and celebrate each of our cultures, languages and beliefs about the natural world and our roles in healing ourselves; to ensure environmental justice; to promote economic alternatives which would contribute to the development of environmentally safe livelihoods; and, to secure our political, economic and cultural liberation that has been denied for over 500 years of colonization and oppression, resulting in the poisoning of our communities and land and the genocide of our peoples, do affirm and adopt these Principles of Environmental Justice” [123].

The introduction to these 17 principles clearly states that environmental justice is and must be comprehensive and effectively address the cumulative effects of racism on the multiple dimensions of the environment mentioned above. Environmental justice matters more so now than ever. It is an important and pervasive issue, especially in today’s climate. If we do not take environmental justice issues seriously, they can have detrimental consequences for thousands of low-income communities and BIPOC across the planet as it relates to the distribution and siting of amenities (e.g., parks; transit service) and disamenities (e.g., industrial facilities). Additionally, there is a growing body of work that shows how climate change, disasters, and critical infrastructure create unequal impacts on BIPOC, impoverished communities, and in low-income countries [124]. For example, climate justice work has begun a discussion on how marginalized groups experience hardships when it comes to the ability to resist and respond to climate change as well as the undue burdens of climate impacts. Across all these different forms of environmental racism, there is an unwaveringly negative impact on the ability of communities to thrive within the human-built-social environment, a place where they live, work, and play, which has profound consequences for everything from public health to generational wealth, particularly in low-income communities and BIPOC.

Achieving environmental justice fundamentally begins with better land use planning and terminating land uses that are incompatible with each other, such as residential and industrial. For future planned industrial sites, we also have to strongly enforce environmental impact assessments and regulate them properly. To confront racism in the field of environmental health, scholars recommend that we develop new measures of racism, consider structural racism as a factor in environmental risk/health assessments, and develop guidelines for the use of race and ethnicity in research [125]. For existing communities, we have to strongly consider equitable buyout programs and dollar amounts for these buyouts that allow homeowners and renters to relocate to other affordable and better quality neighborhoods. There also needs to be regular and triangulated air quality monitoring between residential communities, industries, and municipalities. For communities residing in close proximity to industry or other polluting sources, there should be a provision of medical infrastructure resources for their public health needs and potentially some level of compensation for any liability from the polluting sources as it relates to public health impacts. The suggestion of compensation is not the ultimate solution, but a means to disincentivize harm done by industry, particularly those already located in residential areas. The idea is that by forcing industry to provide resources for public health infrastructure and medical costs, that they would be inclined economically to reduce and minimize impact. Appropriate compensation can be determined by developing and leveraging public health, social, economic (e.g., productivity), and medical studies that show the real cost incurred over a lifetime of morbidity and mortality caused by overexposure to industrial emissions. Ethically, compensation has been used as a form of accountability when harm is done to persons, in and outside of, environmental justice circumstances. Again, compensation is not the goal, but one dimension of accountability. Overall, mitigation is key, good land use planning, comprehensive planning, and smart growth in smart places that is fair, just, and inclusive is essential and imperative.

## Conclusions

*If a set of decision makers believes that an environmental burden can be shouldered by someone else to whom they don’t feel connected or accountable, they won’t think it’s worthwhile to minimize the burden […]*. *But that results in a system that creates more pollution than would exist if decision makers cared about everyone equally* [6].

Heather McGhee

Although this review exemplified environmental racism through an African and/or Black American lens, this type of injustice has had profound impacts on all BIPOC in the USA from past to present. Looking forward toward solutions, some will say that addressing inequality is a zero-sum game. This means that when the lives of some communities of color are improved, other lives, specifically affluent White communities, will then become worse off. Heather McGhee’s book, *The Sum of Us: What Racism Costs Everyone and How We Can Prosper Together*, illustrates the costs of this flawed rationale and way of thinking. While eliminating environmental racism would certainly benefit BIPOC, investing in clean, safe, and healthy environments will ultimately improve the lives for everyone. As long as the false zero-sum narrative prevails, environmental hazards ranging from the contaminated water crisis in Flint, MI, to the police brutality of George Floyd and many others will continue to disproportionately impact low-income communities and BIPOC while also having cascading impacts across society as a whole. In order to achieve environmental justice, we have to understand, acknowledge, and explicitly seek antiracist policies and actions against the multiple dimensions of environmental racism and injustice.
